# Supplementary material for: Safety and Immunogenicity of Trivalent Oral Polio Vaccine in Vaccinated Children and Vaccine-Naïve Infants: A Phase 4 Study
Source: Vaccines (Basel). 2024 Aug 23;12(9):953. doi: 10.3390/vaccines12090953 (PMC11436059; doi:10.3390/vaccines12090953)
Supplement: Supplementary file 1 [file vaccines-12-00953-s001.zip › vaccines-3144026-supplementary.pdf]

Table S1. Incidence of Serious Adverse Events considered consistent with a causal association to study vaccine (SARs), Important Medical Reactions (IMRs), and Severe Adverse Reactions (ARs) by category, time period, and group – TV Population.

AE Type[1]		Group 1 (N=50)	Group 2 Post Dose 1 (N=104)	Group 2 Post Dose 2 (N=104)	Group 2 Post Dose 3 (N=104)	Group 2 (N=104)	Total (N=154)
Subjects with ≥1 SAR, IMR and Severe AR	n (%)	0 (0.0)	1 (1.0)	0 (0.0)	1 (1.0)	2 (1.9)	2 (1.3)
Subjects with ≥1 SAR[2]	n (%)	0 (0.0)	0 (0.0)	0 (0.0)	0 (0.0)	0 (0.0)	0 (0.0)
Subjects with ≥1 IMR[3]	n (%)	0 (0.0)	0 (0.0)	0 (0.0)	0 (0.0)	0 (0.0)	0 (0.0)
Subjects with ≥1 Severe AR[4]	n (%)	0 (0.0)	1 (1.0)	0 (0.0)	1 (1.0)	2 (1.9)	2 (1.3)

Group 1 and Group 2 post 1st dose cannot be combined because of differing follow-up times.

[1] Each subject is counted only once within each AE type.

[2] Within each AE category, a subject may only contribute once.

[3] A subject may only contribute once under the maximum severity observed for any event.

[4] Severe ARs are severe ARs that are not also SARs.

Type 1 poliovirus:

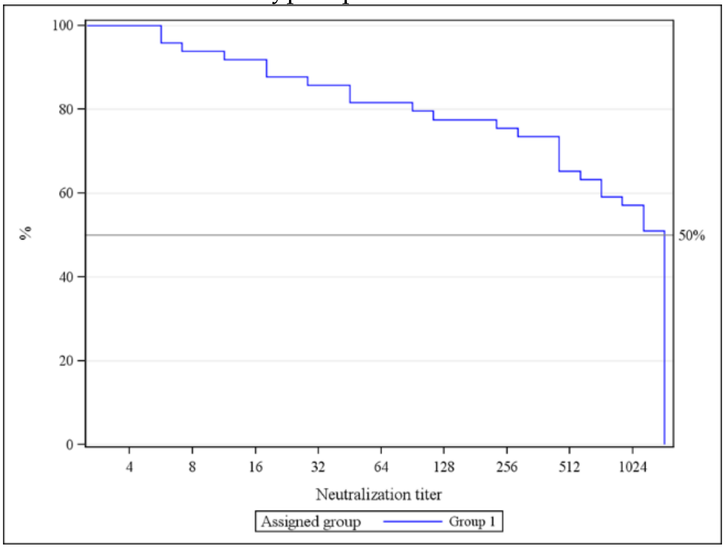

Type 2 poliovirus:

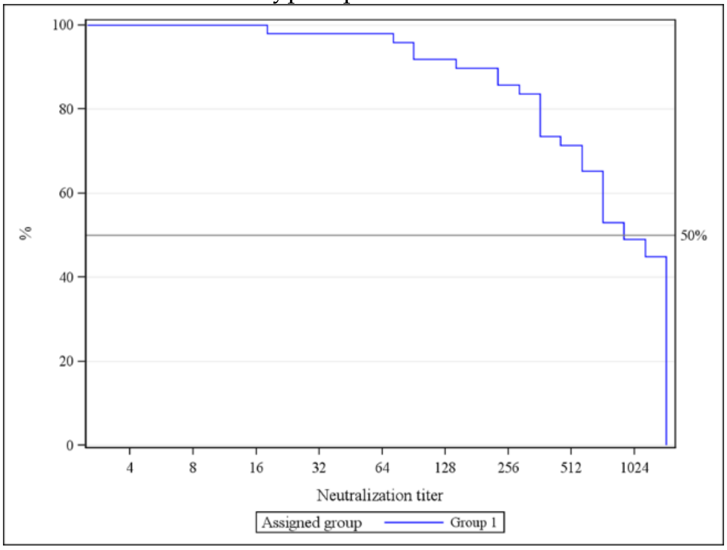

Type 3 poliovirus:

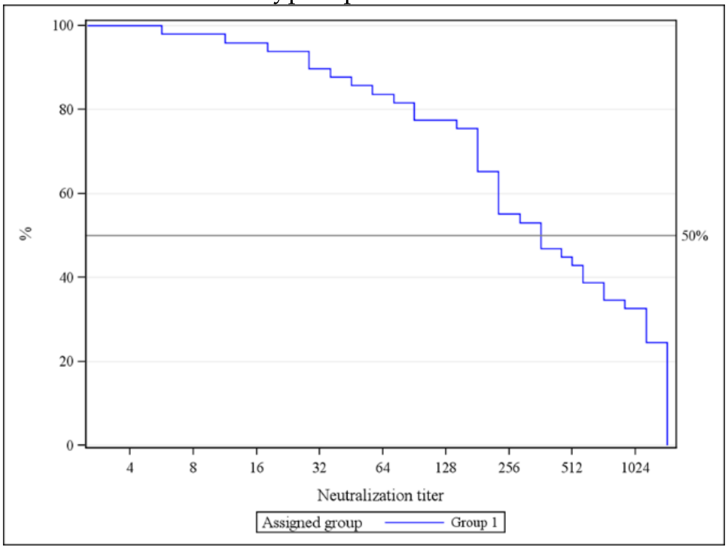

Figure S1. Reverse cumulative distribution plots of poliovirus type-specific neutralizing antibody titers for Group 1 at Day 28 – PP Population.

Type 1 poliovirus:

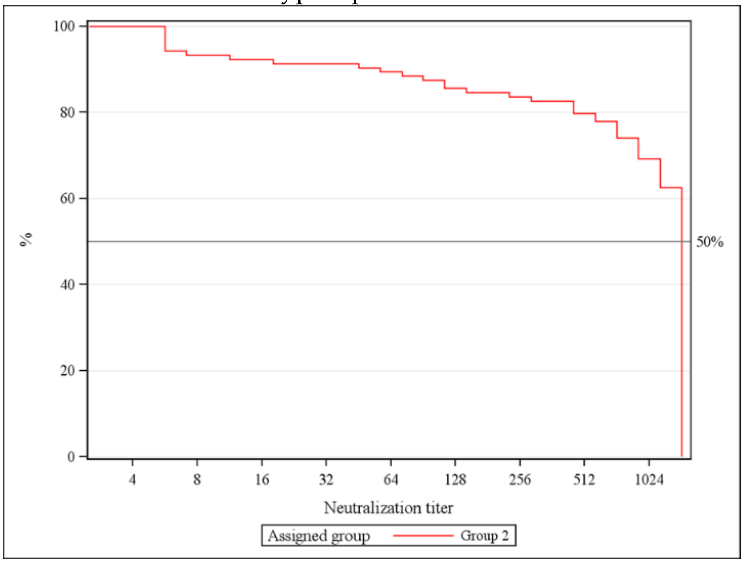

Type 2 poliovirus:

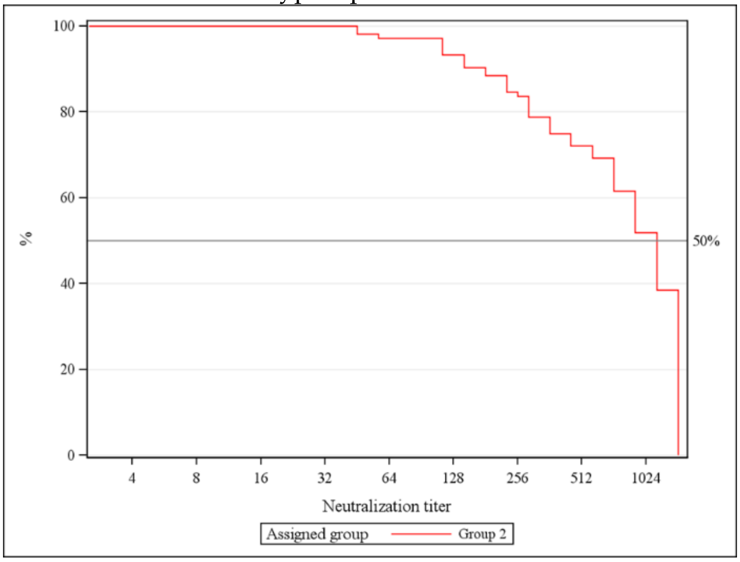

Type 3 poliovirus:

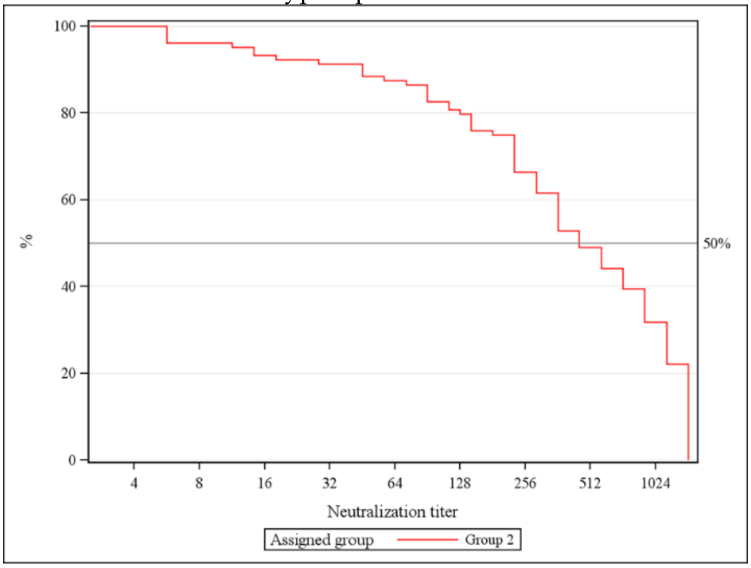

Figure S2. Reverse cumulative distribution plots of poliovirus type-specific neutralizing antibody titers for Group 2 at Day 84 – PP Population.
